# The regulatory mechanisms of NG2/CSPG4 expression

**DOI:** 10.1186/s11658-017-0035-3

**Published:** 2017-02-28

**Authors:** Emmanuel Ampofo, Beate M. Schmitt, Michael D. Menger, Matthias W. Laschke

**Affiliations:** Institute for Clinical & Experimental Surgery, Saarland University, 66421 Homburg, Germany

**Keywords:** NG2, CSPG4, Inflammation, Hypoxia, Methylation, Transcription, MiRNA

## Abstract

Neuron-glial antigen 2 (NG2), also known as chondroitin sulphate proteoglycan 4 (CSPG4), is a surface type I transmembrane core proteoglycan that is crucially involved in cell survival, migration and angiogenesis. NG2 is frequently used as a marker for the identification and characterization of certain cell types, but little is known about the mechanisms regulating its expression. In this review, we provide evidence that the regulation of NG2 expression underlies inflammation and hypoxia and is mediated by methyltransferases, transcription factors, including Sp1, paired box (Pax) 3 and Egr-1, and the microRNA miR129-2. These regulatory factors crucially determine NG2-mediated cellular processes such as glial scar formation in the central nervous system (CNS) or tumor growth and metastasis. Therefore, they are potential targets for the establishment of novel NG2-based therapeutic strategies in the treatment of CNS injuries, cancer and other conditions of these types.

## Introduction

In the last 40 years, many studies have analyzed the structure and functions of neuron-glial antigen 2 (NG2), which is also known as chondroitin sulphate proteoglycan 4 (CSPG4), high molecular weight melanoma-associated antigen (HMW-MAA) or melanoma chondroitin sulfate proteoglycan (MCSP) [1–4]. The NG2 or CSPG4 gene encodes a surface type I transmembrane core protein of ~300 kDa [5]. The extracellular N-terminal domain of this protein is post-translationally modified by chondroitin sulfate glycosaminoglycan chains and disulfide bonds. It also contains putative proteolytic cleavage sites [6]. The function of the extracellular domain fragments is still widely unknown. However, a growing body of evidence suggests that they are involved in the regulation of neuronal networks [7] or endothelial and pericyte functions [8]. The intracellular C-terminal domain of NG2 acts as an acceptor site for the extracellular signal-regulated kinases (ERK) 1/2 and protein kinase C-alpha (PKC-α) as well as a binding site for multi-PZD domain protein 1 (MUPP-1). These interactions activate key signaling pathways involved in cell migration, cell survival and angiogenesis [9, 10].

NG2-mediated signaling has been shown to play an important role in the progression of several tumor types. For instance, elevated NG2 expression is predominantly found in glioblastoma and this correlates with a poor prognosis due to increased NG2-mediated chemo- and radioresistance of the tumor cells [11, 12]. In addition, NG2 serves as a key intermediate of tumor cells with extracellular matrix molecules and thus crucially determines metastatic formation in soft-tissue sarcoma and melanoma patients [13, 14]. Accordingly, NG2 is a promising target for the development of novel tumor therapies [15–17].

NG2 is also expressed in certain benign cell types. In particular, high levels are detected in NG2-glia of the central nervous system (CNS) [18, 19]. NG2-glia are non-neuronal, non-vascular cells that underlie a complex interplay of epigenetic mechanisms and transcription factors in distinct developmental stages [20, 21]. They are sometimes called polydendrocytes because of their branched morphology or oligodendrocyte precursor cells (OPCs) due to their contribution to the renewal and maintenance of the oligodendrocyte population [22, 23]. Mesenchymal stem cells, osteoblasts, melanocytes, smooth muscle cells and macrophages have also been shown to express NG2 [3, 24, 25]. Finally, the proteoglycan is a typical marker for vessel-surrounding pericytes, which contribute to the stabilization of microvessels, the regulation of capillary blood flow and angiogenesis [26]. Interestingly, the expression pattern of NG2 markedly differs between distinct pericytes depending on the type of analyzed tissue. For instance, only arteriolar but not venular pericytes are positive for NG2 in the mesenteric microvascular network [27, 28]. By contrast, the proteoglycan is expressed in pericytes of all of the microvascular segments in the retina: arterioles, capillaries and venules [29].

These findings indicate that the expression of NG2 underlies a finely balanced regulation dependent on specific cell functions in different tissues. However, which factors are involved in this regulation and how they interact with each other remains elusive. As outlined in the following review, NG2 expression is influenced by inflammation and hypoxia and is intracellularly regulated by methyltransferases, transcription factors and miRNAs (Table 1).

**Table 1:** Studies focusing on factors which regulate the expression of NG2/CSPG4

Regulatory factors / treatment		Species	Type of cells or tissue	Analysis	Expression	Reference
Inflammation	TNF-α, TGF-β, IL-1α or IFNγ	Rat	OPC	Protein	↑	[22]
	TGF-β	Mouse	Macrophages, OPC	mRNA, Protein	↑	[24]
	TGF-β	Rat	Cerebral cortex	Protein	↑	[25]
	Decorin	Rat	Spinal cord	Protein	↓	[26]
	TGF-β receptor inhibitor	Rat	Microglia cells	mRNA, Protein	↓	[28]
	LPS	Rat	Microglia cells	mRNA, Protein	↑	[23]
	IL11, LIF	Human	Placental villous tissue	mRNA, Protein	↑	[32]
	Chronic hypoxia (5 d)	Rat	Mesentery	Protein	↑	[35]
Hypoxia	Chronic hypoxia (48 h)	Human	Panc1, HS766T	mRNA, Protein	↑	[39]
DNA Methylation	Methyltransferase inhibitor (5-aza-2’-deoxycytidine)	Human	Melanoma cells	mRNA, Protein	↑	[45]
	Methyltransferase inhibitor (5-aza-2’-deoxycytidine)	Human	Head and neck squamous cell carcinoma	mRNA, Protein	↑	[46]
Transcription factors	Truncated promoter constructs	Monkey	COS cells	Luciferase activity	↑	[49]
	Sp1 siRNA	Human	Keratinocytes	mRNA	↓	[50]
	Pax3	Human	Melanocytes, melanoma cells	mRNA	↑	[54]
	Pax3 siRNA	Human	Melanocytes, melanoma cells	mRNA	↓	[55]
	Egr1 siRNA	Mouse/Human	Astrocytes	mRNA, Protein	↓	[31]
miRNA	miR129-2	Mouse	Neurospheres	mRNA, Protein	↓	[62]

The table lists the species and type of cells or tissue in which the analyses have been performed as well as the level of detection and the observed up- (↑) or downregulation (↓) of NG2/CSPG4 expression

### Inflammation

Different NG2-positive cell types in the CNS express receptors for inflammatory cytokines [30–32]. Studies indicate that these cytokines are directly involved in the regulation of NG2 expression and function in response to CNS injuries. For instance, disruption of the blood–brain barrier has been shown to stimulate the release of tumor necrosis factor-alpha (TNF-α), interleukin 1-alpha (IL-1α) and interferon-gamma (IFN-γ) from platelets and other blood components, resulting in increased NG2 levels in OPCs [33]. Gao et al. [34] further detected a higher mRNA and protein level of NG2 in microglial cells following stimulation with lipopolysaccharide (LPS). This was associated with a higher expression of inducible nitric oxide synthase (iNOS), IL-1β and TNF-α, which could be reversed by treatment with NG2 siRNA. In addition, neuroinflammatory disorders, such as autoimmune encephalomyelitis, elevate the expression of NG2 in OPCs, macrophages and CNS-resident microglia, which is mediated by transforming growth factor-beta (TGF-β) [35, 36]. Inhibition of TFG-β activity by decorin [37, 38] or TGF-β1 receptor signaling by SB525334 attenuates this TGF-β-induced NG2 expression [39].

Taken together, these studies indicate that cytokine-mediated NG2 expression is a major response mechanism to various destructive processes in the CNS. In this context, it should be considered that NG2 is an important contributor to glial scar formation [40], during which increased expression levels of extracellular matrix components and chondroitin sulfate proteoglycans, including NG2, form an inhibitory barrier to regenerating axons, blocking their outgrowth in the surrounding tissue [41, 42]. Accordingly, the modulation of this process may be a promising approach to promote neuronal repair after traumatic or inflammation-induced CNS injuries.

In addition, van Sinderen et al. [43] recently analyzed the role of NG2 in the placenta and extravillous trophoblasts. They found that IL-11 and leukemia inhibitory factor (LIF), known to be produced by the placenta in the first trimester [44, 45], stimulate NG2 expression specifically in the placental villi and deciduas. They speculated that these two cytokines stimulate the early differentiation of the cytotrophoblast cells towards the migratory extravillous trophoblast phenotype via the upregulation of NG2 levels.

### Hypoxia

Several studies show that NG2 expression may be regulated by hypoxia-induced signal transduction. Under normoxic conditions, NG2 expression is only found in pericytes located along the arterioles and capillaries but not along the venules of adult rat mesenteric microvascular networks [28]. Exposure of these networks to chronic hypoxia is associated with additional expression of the proteoglycan in venular pericytes [46]. This indicates an important function of NG2 in these activated cells during hypoxia-induced angiogenesis and vascular remodeling [46]. Concurrent with this view, Ozerdem et al. [47] found a substantially reduced neovascularization in the ischemic retinas of NG2 knockout mice when compared to wild-type controls.

Hypoxia-inducible factors (HIFs) are the most important transcription factors mediating hypoxic expression of target genes [48]. Accordingly, they may also be involved in the regulation of NG2. Under normoxia, HIFs are constitutively expressed in the cytoplasm with a very short half-life, because they are hydroxylated. This promotes their binding to von Hippel-Lindau protein (VHL), which targets HIFs for rapid proteasomal degradation. Under hypoxia, non-hydroxylated HIFs translocate to the nucleus, resulting in increased target gene expression [49]. Concurrent with these findings, Keleg et al. [50] could demonstrate that NG2 mRNA and protein levels are already overexpressed in the normoxic pancreatic cancer cell lines Panc1 and HS766T, which exhibit mutations of VHL. Exposure of these cells to chronic hypoxia further enhances these high NG2 mRNA and protein expression levels.

### DNA methylation

DNA methylation has recently been identified as a major epigenetic mechanism for the regulation of gene expression [51]. It is characterized by the transfer of a methyl group to the 5’ cytosine of a CpG dinucleotide by DNA methyltransferases, resulting in the suppression of gene transcription [52]. Promoter methylation of tumor suppressor genes is particularly found during carcinogenesis, indicating that this process induces the development of many types of tumor [53–55]. Interestingly, Luo et al. [56] found that the promoter of the NG2 gene also contains many 5’CpG methylation sites. It has further been demonstrated that reduced methylation of this promoter increases the expression of NG2 in melanoma cell lines, primary melanoma lesions, and head and neck squamous cell carcinoma [56, 57]. Since high NG2 expression is often associated with elevated multi-drug resistance [11, 12, 58], methylation of the NG2 promoter may thus determine the therapy response and prognosis of many cancer types.

### Transcription factors

In addition to DNA methylation, NG2 expression is also regulated by several transcription factors. In 2009, Sellers et al. [59] described the regulatory region (1585 bp) upstream of the mouse NG2 coding sequence in detail. The TATA-box of the NG2 promoter is 1299 bp upstream of the transcriptional start. Putative binding sites for transcription factors are located within this region. As identified by Transcriptional Element System Search (TESS), these transcription factors may include C/EBP, p300, CBP and Sp1. The latter seems to have a particularly crucial role in the regulation of NG2 gene expression. Sellers et al. [59] showed that transfection of COS cells with luciferase reporter gene constructs that contain the NG2 promoter without a TATA-box and the upstream located Sp1 binding sites, results in increased cellular luciferase activity. On the other hand, Leung et al. [60] found that silencing Sp1 downregulates the transcription of the proteoglycan in keratinocytes. These contradictory findings suggest that additional post-transcriptional modifications of the constitutively expressed transcription factor Sp1 determine its function as a transcriptional activator or repressor of NG2 [61].

Paired box 3 (Pax 3) is another transcription factor capable of influencing target gene transcription in a positive [62] or negative [63] manner. However, Pax3 has only been shown to increase the expression of NG2 in melanocytes [64]. Pax3 silencing results in a diminished expression of the proteoglycan in these cells [65]. It has further been reported that TGF-β suppresses the expression of Pax3 in melanocytes via a smad-dependent pathway [66]. Since this growth factor upregulates NG2 levels in CNS-resident microglia [35, 36, 39], this finding indicates that other transcription factors must be involved in TGF-β-induced NG2 expression or that its regulation markedly differs between individual cell types.

The transcription factor Egr-1 is a crucial mediator in ERK-dependent signaling during cell survival, apoptosis and differentiation [67]. ERK phosphorylates and activates the transcription factor Elk-1 [68, 69], which increases the expression of Egr-1 [70]. Egr-1 regulates the expression of different genes encoding for adhesion proteins and cytokines [71]. Beck et al. [42] demonstrated that after cerebral ischemia, reactive astrocytes exhibit high levels of Egr-1. Silencing Egr-1 in these cells diminished the expression of genes that are important for glial scar formation, including NG2. They concluded that this transcription factor may represent a potential target for the modulation of neuronal tissue repair and regeneration.

### miRNAs

In the last decade, microRNAs (miRNAs) have been identified as novel, powerful regulators of protein expression. They are endogenously expressed small non-coding RNA molecules that suppress protein expression by interacting with the target messenger RNA (mRNA) [52]. miR129-2 belongs to the group of tumor suppressor miRNAs, because its transcription is downregulated in some types of cancer due to increased methylation [72]. It was recently reported that overexpression of miR129-2 decreases NG2 levels in diffuse intrinsic pontine gliomas [73]. miR129-2 also suppresses the expression of platelet-derived growth factor receptor-alpha (PDGFR-α) in glioma cells [74]. This receptor stimulates the proliferation of various mesenchymal and glial cells and is one of the most amplified genes in glioblastoma [75]. In addition, interaction of NG2 with PDGFR-α has been shown to promote cell proliferation in response to PDGF [76, 77]. These findings suggest that miR129-2 may represent a promising candidate for NG2-targeting tumor therapy.

## Conclusion

**Fig. 1:**
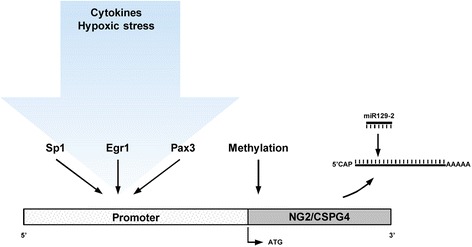
Known regulatory factors of NG2 (CSPG4) expression. NG2 expression underlies inflammatory cytokines and hypoxic stress and is mediated by methyltransferases, transcription factors such as Sp1, Egr1 and Pax3, and miR129-2

Although NG2 is important for cell function and frequently used as a marker for the characterization and identification of certain cell types, there is little knowledge about the mechanisms that regulate the expression of this proteoglycan. The reports discussed here provide the first evidence that this regulation underlies inflammation and hypoxia and is mediated by methyltransferases, transcription factors and miRNAs (Fig. 1). In the future, the identification of additional regulatory factors may further improve our understanding of NG2-mediated cellular functions, such as cell survival, migration and angiogenesis. In addition, it may also contribute to the establishment of NG2 as a novel therapeutic target in the treatment of CNS injuries, cancer and other conditions.

## Abbreviations

CNS: Central nervous system
CSPG4: Chondroitin sulphate proteoglycan 4
ERK: Extracellular-signal-regulated kinase
HIF: Hypoxia-inducible factor
HMW-MAA: High molecular weight melanoma-associated antigen
IFN: Interferon
IL: Interleukin
iNOS: Inducible nitric oxide synthase
LIF: Leukemia inhibitory factor
LPS: Lipopolysaccharide
MCSP: Melanoma chondroitin sulfate proteoglycan
miRNA: MicroRNA
MUPP: Multi-PZD domain protein
NG2/CSPG4: Neuron-glial antigen 2
OPC: Oligodendrocyte precursor cell
Pax: Paired box
PDGFR: Platelet-derived growth factor receptor
PKC: Protein kinase C
TESS: Transcriptional element system search
TGF: Transforming growth factor
TNF: Tumor necrosis factor
VHL: Von Hippel-Lindau.

## References

[1] Pankova D, Jobe N, Kratochvilova M, Buccione R, Brabek J, Rosel D (2012). NG2-mediated Rho activation promotes amoeboid invasiveness of cancer cells. Eur J Cell Biol..

[2] Stallcup WB (2002). The NG2 proteoglycan: past insights and future prospects. J Neurocytol..

[3] Stallcup WB, You WK, Kucharova K, Cejudo-Martin P, Yotsumoto F (2016). NG2 Proteoglycan-Dependent Contributions of Pericytes and Macrophages to Brain Tumor Vascularization and Progression. Microcirculation..

[4] Wilson BS, Ruberto G, Ferrone S (1983). Immunochemical characterization of a human high molecular weight--melanoma associated antigen identified with monoclonal antibodies. Cancer Immunol Immunother..

[5] Pluschke G, Vanek M, Evans A, Dittmar T, Schmid P, Itin P, Filardo EJ, Reisfeld RA (1996). Molecular cloning of a human melanoma-associated chondroitin sulfate proteoglycan. Proc Natl Acad Sci U S A..

[6] Yadavilli S, Hwang EI, Packer RJ, Nazarian J (2016). The Role of NG2 Proteoglycan in Glioma. Transl Oncol..

[7] Sakry D, Neitz A, Singh J, Frischknecht R, Marongiu D, Biname F, Perera SS, Endres K, Lutz B, Radyushkin K (2014). Oligodendrocyte precursor cells modulate the neuronal network by activity-dependent ectodomain cleavage of glial NG2. PLoS Biol..

[8] You WK, Yotsumoto F, Sakimura K, Adams RH, Stallcup WB (2014). NG2 proteoglycan promotes tumor vascularization via integrin-dependent effects on pericyte function. Angiogenesis..

[9] Makagiansar IT, Williams S, Mustelin T, Stallcup WB (2007). Differential phosphorylation of NG2 proteoglycan by ERK and PKCalpha helps balance cell proliferation and migration. J Cell Biol..

[10] Barritt DS, Pearn MT, Zisch AH, Lee SS, Javier RT, Pasquale EB, Stallcup WB (2000). The multi-PDZ domain protein MUPP1 is a cytoplasmic ligand for the membrane-spanning proteoglycan NG2. J Cell Biochem..

[11] Svendsen A, Verhoeff JJ, Immervoll H, Brogger JC, Kmiecik J, Poli A, Netland IA, Prestegarden L, Planaguma J, Torsvik A (2011). Expression of the progenitor marker NG2/CSPG4 predicts poor survival and resistance to ionising radiation in glioblastoma. Acta Neuropathol..

[12] Chekenya M, Krakstad C, Svendsen A, Netland IA, Staalesen V, Tysnes BB, Selheim F, Wang J, Sakariassen PO, Sandal T (2008). The progenitor cell marker NG2/MPG promotes chemoresistance by activation of integrin-dependent PI3K/Akt signaling. Oncogene..

[13] Mittelman A, Chen ZJ, Liu CC, Hirai S, Ferrone S (1994). Kinetics of the immune response and regression of metastatic lesions following development of humoral anti-high molecular weight-melanoma associated antigen immunity in three patients with advanced malignant melanoma immunized with mouse antiidiotypic monoclonal antibody MK2-23. Cancer Res..

[14] Benassi MS, Pazzaglia L, Chiechi A, Alberghini M, Conti A, Cattaruzza S, Wassermann B, Picci P, Perris R (2009). NG2 expression predicts the metastasis formation in soft-tissue sarcoma patients. J Orthop Res..

[15] Pucciarelli D, Lengger N, Takacova M, Csaderova L, Bartosova M, Breiteneder H, Pastorekova S, Hafner C (2015). Anti-chondroitin sulfate proteoglycan 4-specific antibodies modify the effects of vemurafenib on melanoma cells differentially in normoxia and hypoxia. Int J Oncol..

[16] Guan YY, Luan X, Xu JR, Liu YR, Lu Q, Wang C, Liu HJ, Gao YG, Chen HZ, Fang C (2014). Selective eradication of tumor vascular pericytes by peptide-conjugated nanoparticles for antiangiogenic therapy of melanoma lung metastasis. Biomaterials..

[17] Falvo E, Tremante E, Fraioli R, Leonetti C, Zamparelli C, Boffi A, Morea V, Ceci P, Giacomini P (2013). Antibody-drug conjugates: targeting melanoma with cisplatin encapsulated in protein-cage nanoparticles based on human ferritin. Nanoscale..

[18] Nishiyama A, Boshans L, Goncalves CM, Wegrzyn J, Patel KD (2016). Lineage, fate, and fate potential of NG2-glia. Brain Res.

[19] Sakry D, Trotter J (2016). The role of the NG2 proteoglycan in OPC and CNS network function. Brain Res.

[20] Küspert M, Wegner M (2016). SomethiNG 2 talk about-Transcriptional regulation in embryonic and adult oligodendrocyte precursors. Brain Res.

[21] Moyon S, Liang J, Casaccia P (2016). Epigenetics in NG2 glia cells. Brain Res.

[22] Eugenín-von Bernhardi J, Dimou L (2016). NG2-glia, More Than Progenitor Cells. Adv Exp Med Biol..

[23] Viganò F, Dimou L (2016). The heterogeneous nature of NG2-glia. Brain Res.

[24] Nishiyama A, Komitova M, Suzuki R, Zhu X (2009). Polydendrocytes (NG2 cells): multifunctional cells with lineage plasticity. Nat Rev Neurosci..

[25] Hughes S, Chan-Ling T (2004). Characterization of smooth muscle cell and pericyte differentiation in the rat retina in vivo. Invest Ophthalmol Vis Sci..

[26] Trost A, Lange S, Schroedl F, Bruckner D, Motloch KA, Bogner B, Kaser-Eichberger A, Strohmaier C, Runge C, Aigner L (2016). Brain and Retinal Pericytes: Origin, Function and Role. Front Cell Neurosci.

[27] Alon R, Nourshargh S (2013). Learning in motion: pericytes instruct migrating innate leukocytes. Nat Immunol..

[28] Murfee WL, Skalak TC, Peirce SM (2005). Differential arterial/venous expression of NG2 proteoglycan in perivascular cells along microvessels: identifying a venule-specific phenotype. Microcirculation..

[29] Chan-Ling T, Hughes S (2005). NG2 can be used to identify arteries versus veins enabling the characterization of the different functional roles of arterioles and venules during microvascular network growth and remodeling. Microcirculation..

[30] Dopp JM, Mackenzie-Graham A, Otero GC, Merrill JE (1997). Differential expression, cytokine modulation, and specific functions of type-1 and type-2 tumor necrosis factor receptors in rat glia. J Neuroimmunol..

[31] Friedman WJ (2001). Cytokines regulate expression of the type 1 interleukin-1 receptor in rat hippocampal neurons and glia. Exp Neurol..

[32] Colton CA (2009). Heterogeneity of microglial activation in the innate immune response in the brain. J Neuroimmune Pharmacol..

[33] Rhodes KE, Raivich G, Fawcett JW (2006). The injury response of oligodendrocyte precursor cells is induced by platelets, macrophages and inflammation-associated cytokines. Neuroscience..

[34] Gao Q, Lu J, Huo Y, Baby N, Ling EA, Dheen ST (2010). NG2, a member of chondroitin sulfate proteoglycans family mediates the inflammatory response of activated microglia. Neuroscience..

[35] Moransard M, Dann A, Staszewski O, Fontana A, Prinz M, Suter T (2011). NG2 expressed by macrophages and oligodendrocyte precursor cells is dispensable in experimental autoimmune encephalomyelitis. Brain..

[36] Xiang P, Zhu L, Jiang H, He BP (2015). The activation of NG2 expressing cells is downstream to microglial reaction and mediated by the transforming growth factor beta 1. J Neuroimmunol..

[37] Davies JE, Tang X, Denning JW, Archibald SJ, Davies SJ (2004). Decorin suppresses neurocan, brevican, phosphacan and NG2 expression and promotes axon growth across adult rat spinal cord injuries. Eur J Neurosci..

[38] Yamaguchi Y, Mann DM, Ruoslahti E (1990). Negative regulation of transforming growth factor-beta by the proteoglycan decorin. Nature..

[39] Sugimoto K, Nishioka R, Ikeda A, Mise A, Takahashi H, Yano H, Kumon Y, Ohnishi T, Tanaka J (2014). Activated microglia in a rat stroke model express NG2 proteoglycan in peri-infarct tissue through the involvement of TGF-beta1. Glia..

[40] Tan AM, Zhang W, Levine JM (2005). NG2: a component of the glial scar that inhibits axon growth. J Anat..

[41] Silver J (2016). The glial scar is more than just astrocytes. Exp Neurol..

[42] Beck H, Semisch M, Culmsee C, Plesnila N, Hatzopoulos AK (2008). Egr-1 regulates expression of the glial scar component phosphacan in astrocytes after experimental stroke. Am J Pathol..

[43] Van Sinderen M, Cuman C, Winship A, Menkhorst E, Dimitriadis E (2013). The chrondroitin sulfate proteoglycan (CSPG4) regulates human trophoblast function. Placenta..

[44] Suman P, Shembekar N, Gupta SK (2013). Leukemia inhibitory factor increases the invasiveness of trophoblastic cells through integrated increase in the expression of adhesion molecules and pappalysin 1 with a concomitant decrease in the expression of tissue inhibitor of matrix metalloproteinases. Fertil Steril..

[45] Paiva P, Salamonsen LA, Manuelpillai U, Walker C, Tapia A, Wallace EM, Dimitriadis E (2007). Interleukin-11 promotes migration, but not proliferation, of human trophoblast cells, implying a role in placentation. Endocrinology..

[46] Murfee WL, Rehorn MR, Peirce SM, Skalak TC (2006). Perivascular cells along venules upregulate NG2 expression during microvascular remodeling. Microcirculation..

[47] Ozerdem U, Stallcup WB (2004). Pathological angiogenesis is reduced by targeting pericytes via the NG2 proteoglycan. Angiogenesis..

[48] Bardos JI, Ashcroft M (2005). Negative and positive regulation of HIF-1: a complex network. Biochim Biophys Acta.

[49] Baldewijns MM, van Vlodrop IJ, Vermeulen PB, Soetekouw PM, van Engeland M, de Bruine AP (2010). VHL and HIF signalling in renal cell carcinogenesis. J Pathol..

[50] Keleg S, Titov A, Heller A, Giese T, Tjaden C, Ahmad SS, Gaida MM, Bauer AS, Werner J, Giese NA (2014). Chondroitin sulfate proteoglycan CSPG4 as a novel hypoxia-sensitive marker in pancreatic tumors. PLoS One..

[51] Akhavan-Niaki H, Samadani AA (2013). DNA methylation and cancer development: molecular mechanism. Cell Biochem Biophys..

[52] Herman JG, Baylin SB (2003). Gene silencing in cancer in association with promoter hypermethylation. N Engl J Med..

[53] Hasegawa M, Nelson HH, Peters E, Ringstrom E, Posner M, Kelsey KT (2002). Patterns of gene promoter methylation in squamous cell cancer of the head and neck. Oncogene..

[54] Kulkarni V, Saranath D (2004). Concurrent hypermethylation of multiple regulatory genes in chewing tobacco associated oral squamous cell carcinomas and adjacent normal tissues. Oral Oncol..

[55] Yeh KT, Shih MC, Lin TH, Chen JC, Chang JY, Kao CF, Lin KL, Chang JG (2002). The correlation between CpG methylation on promoter and protein expression of E-cadherin in oral squamous cell carcinoma. Anticancer Res..

[56] Luo W, Wang X, Kageshita T, Wakasugi S, Karpf AR, Ferrone S (2006). Regulation of high molecular weight-melanoma associated antigen (HMW-MAA) gene expression by promoter DNA methylation in human melanoma cells. Oncogene..

[57] Warta R, Herold-Mende C, Chaisaingmongkol J, Popanda O, Mock A, Mogler C, Osswald F, Herpel E, Kustner S, Eckstein V (2014). Reduced promoter methylation and increased expression of CSPG4 negatively influences survival of HNSCC patients. Int J Cancer..

[58] Schrappe M, Klier FG, Spiro RC, Waltz TA, Reisfeld RA, Gladson CL (1991). Correlation of chondroitin sulfate proteoglycan expression on proliferating brain capillary endothelial cells with the malignant phenotype of astroglial cells. Cancer Res..

[59] Sellers DL, Maris DO, Horner PJ (2009). Postinjury niches induce temporal shifts in progenitor fates to direct lesion repair after spinal cord injury. J Neurosci..

[60] Bin L, Kim BE, Hall CF, Leach SM, Leung DY (2011). Inhibition of transcription factor specificity protein 1 alters the gene expression profile of keratinocytes leading to upregulation of kallikrein-related peptidases and thymic stromal lymphopoietin. J Invest Dermatol..

[61] Tan NY, Khachigian LM (2009). Sp1 phosphorylation and its regulation of gene transcription. Mol Cell Biol..

[62] Watanabe A, Takeda K, Ploplis B, Tachibana M (1998). Epistatic relationship between Waardenburg syndrome genes MITF and PAX3. Nat Genet..

[63] Li HG, Wang Q, Li HM, Kumar S, Parker C, Slevin M, Kumar P (2007). PAX3 and PAX3-FKHR promote rhabdomyosarcoma cell survival through downregulation of PTEN. Cancer Lett..

[64] Medic S, Rizos H, Ziman M (2011). Differential PAX3 functions in normal skin melanocytes and melanoma cells. Biochem Biophys Res Commun..

[65] Bartlett D, Boyle GM, Ziman M, Medic S (2015). Mechanisms contributing to differential regulation of PAX3 downstream target genes in normal human epidermal melanocytes versus melanoma cells. PLoS One..

[66] Yang G, Li Y, Nishimura EK, Xin H, Zhou A, Guo Y, Dong L, Denning MF, Nickoloff BJ, Cui R (2008). Inhibition of PAX3 by TGF-beta modulates melanocyte viability. Mol Cell..

[67] McCain J (2013). The MAPK (ERK) Pathway: Investigational Combinations for the Treatment Of BRAF-Mutated Metastatic Melanoma. P T.

[68] Gille H, Kortenjann M, Thomae O, Moomaw C, Slaughter C, Cobb MH, Shaw PE (1995). ERK phosphorylation potentiates Elk-1-mediated ternary complex formation and transactivation. EMBO J..

[69] Janknecht R, Ernst WH, Pingoud V, Nordheim A (1993). Activation of ternary complex factor Elk-1 by MAP kinases. EMBO J..

[70] Gregg J, Fraizer G (2011). Transcriptional Regulation of EGR1 by EGF and the ERK Signaling Pathway in Prostate Cancer Cells. Genes Cancer..

[71] Svaren J, Ehrig T, Abdulkadir SA, Ehrengruber MU, Watson MA, Milbrandt J (2000). EGR1 target genes in prostate carcinoma cells identified by microarray analysis. J Biol Chem..

[72] Banno K, Yanokura M, Iida M, Masuda K, Aoki D (2014). Carcinogenic mechanisms of endometrial cancer: involvement of genetics and epigenetics. J Obstet Gynaecol Res..

[73] Yadavilli S, Scafidi J, Becher OJ, Saratsis AM, Hiner RL, Kambhampati M, Mariarita S, MacDonald TJ, Codispoti KE, Magge SN (2015). The emerging role of NG2 in pediatric diffuse intrinsic pontine glioma. Oncotarget..

[74] Tian XY, Zhang L, Sun LG, Li M (2015). Epigenetic Regulation of miR-129-2 Leads to Overexpression of PDGFRa and FoxP1 in Glioma Cells. Asian Pac J Cancer Prev..

[75] Liu KW, Hu B, Cheng SY (2011). Platelet-derived growth factor receptor alpha in glioma: a bad seed. Chin J Cancer..

[76] Grako KA, Ochiya T, Barritt D, Nishiyama A, Stallcup WB (1999). PDGF (alpha)-receptor is unresponsive to PDGF-AA in aortic smooth muscle cells from the NG2 knockout mouse. J Cell Sci.

[77] Nishiyama A, Lin XH, Giese N, Heldin CH, Stallcup WB (1996). Interaction between NG2 proteoglycan and PDGF alpha-receptor on O2A progenitor cells is required for optimal response to PDGF. J Neurosci Res..

